# Safe and efficient intracellular delivery of functional antibodies into cultured neural cells shows a role for Alzheimer’s-relevant tau phosphorylation in cell integrity

**DOI:** 10.3389/frdem.2026.1737082

**Published:** 2026-10-13

**Authors:** Kirsten L. Viola, Brett A. Chromy, Raghad Nowar, Mary P. Lambert, William L. Klein

**Affiliations:** Department of Neurobiology, Northwestern University, Evanston, IL, United States

**Keywords:** cationic liposomes, intracellular, neurodegeneration, PHF-1 antibody, tau

## Abstract

**Introduction:**

Protein proteoforms generated by post-translational modification are increasingly recognized as important regulators of neuronal function and neurodegenerative disease. However, methods for acutely interrogating specific intracellular proteoforms in living neuronal populations remain limited. We evaluated cationic liposomal delivery (CLD) as a strategy for introducing full-length functional antibodies into living neurons and used this approach to investigate the role of a phosphorylated tau proteoform recognized by the monoclonal antibody PHF-1.

**Methods:**

Cationic liposomes composed of DOPC+ were complexed with antibodies or molecular probes and applied to cultured neuronal cell lines and primary mouse cortical neurons. Intracellular delivery, cellular localization, viability, and biological activity were assessed using fluorescence microscopy and live-cell imaging.

**Results:**

CLD efficiently delivered full-length antibodies and molecular probes into living neuronal cells and primary neurons without detectable toxicity. Internalized antibodies retained target specificity, producing intracellular staining patterns consistent with the known localization of tau, vinculin, paxillin, and Fyn. Delivery was observed across essentially all cells in treated cultures and remained effective under standard culture conditions. Intracellular delivery of PHF-1 produced a rapid and striking morphological response characterized by neurite retraction and cell-body rounding within hours of treatment. These effects were not observed with heat-inactivated PHF-1, vehicle controls, or other intracellular antibodies, supporting a specific effect of intracellular engagement of PHF-1-reactive tau species.

**Discussion:**

These findings establish CLD as a practical platform for the intracellular delivery of functional full-length antibodies into living neuronal populations. Beyond validating a delivery technology, the study demonstrates that acute intracellular engagement of PHF-1-reactive phosphorylated tau rapidly alters neuronal morphology, suggesting that this tau proteoform contributes to the maintenance of neurite integrity. CLD provides a versatile approach for probing the function of intracellular proteoforms that are difficult to study using conventional genetic or biochemical methods.

## 1 Introduction

Post-translational modification generates distinct protein proteoforms with different—and sometimes opposing—biological activities. This complexity is especially important in neurodegenerative disease, where disease-associated proteoforms may coexist with closely related species that perform essential physiological functions. Tau provides a prominent example. In Alzheimer’s disease (AD) and related tauopathies, abnormal tau phosphorylation is associated with impaired microtubule interactions, altered intracellular signaling, misfolding and self-assembly, and ultimately formation of neurofibrillary tangles (NFTs) (Braak and Braak, 1991; Wang and Mandelkow, 2016). Tau pathology is strongly associated with synaptic dysfunction, neuronal loss, and cognitive decline, making disease-associated tau proteoforms important mechanistic and therapeutic targets (Kosik et al., 1989; Takashima, 2010; Goedert and Spillantini, 2011; Medeiros et al., 2011; Irwin et al., 2012; Rawat et al., 2022; Chen and Yu, 2023; Goedert et al., 2024).

Advances in proteomics and phosphoproteomics have increasingly made it possible to define disease-associated proteins at the proteoform level, revealing specific tau phosphorylation patterns and candidate epitopes that may distinguish biologically important tau species (Barthelemy et al., 2020; Ashton et al., 2021; Fang et al., 2024; Mammeri et al., 2024; Fulcher et al., 2025; Xu et al., 2025; Kumar et al., 2026; Vijayalakshmi and Prabhakar, 2026). A major experimental challenge, however, is determining the functions of individual proteoforms in living cells. Conventional genetic manipulations generally alter expression of the entire protein rather than acutely perturbing a selected post-translationally modified species. Methods capable of selectively engaging individual proteoforms in intact cells therefore could provide powerful tools for distinguishing their physiological and pathological functions.

Antibodies are particularly attractive for this purpose because they can discriminate among protein conformations, aggregation states, and post-translational modifications with exceptional specificity. Intracellular antibodies and genetically encoded intrabodies can alter protein interactions, aggregation, trafficking, and degradation (Miller and Messer, 2005; Gallardo et al., 2019), and anti-tau intrabodies have reduced tau pathology in experimental models (Gallardo et al., 2019; Goodwin et al., 2021; Rodrigues Martins et al., 2023). Their broader experimental use, however, is constrained by a fundamental problem: intact antibodies do not readily cross the plasma membrane and, following uptake, may remain trapped within endosomal compartments rather than gaining access to cytoplasmic targets (Slastnikova et al., 2018; Chan and Tsourkas, 2024; Zhu et al., 2024). A rapid, minimally disruptive method for introducing functional full-length antibodies into populations of living neurons therefore would provide a useful alternative to genetically encoded intrabodies and other approaches requiring prolonged or invasive manipulation.

Cationic liposomes offer one potential solution. Liposomes formed from 1,2-dioleoyl-sn-glycero-3-ethylphosphocholine (DOPC+) have previously been used to transfer membrane-impermeant probes and other cargo into living cells with relatively low toxicity (Barber et al., 1996; MacDonald et al., 1999b; Pantazatos and MacDonald, 1999; Shim et al., 2011; Wang and MacDonald, 2011). More broadly, cationic lipid systems can transport proteins and antibodies intracellularly while preserving biological activity (Wang and Tsourkas, 2019). These properties suggested that DOPC+-mediated cationic liposomal delivery (CLD) might provide a simple means of introducing functional antibodies into entire populations of living neuronal cells and thereby permit acute perturbation of selected intracellular proteoforms.

We examined this possibility using PHF-1, a well-characterized monoclonal antibody that recognizes tau phosphorylated at Ser396 and Ser404 (Bodenteich et al., 1992; Otvos Jr et al., 1994; Augustinack et al., 2002; Henkins et al., 2012). Although PHF-1 reactivity is strongly associated with pathological tau in AD, phosphorylation at these and related epitopes is not exclusively pathological. PHF-1-reactive tau also appears during normal neuronal development, when tau phosphorylation is dynamically regulated in association with neuronal differentiation and process formation (Brion et al., 1993; Goedert et al., 1993; Kenessey and Yen, 1993a; Pope et al., 1993; Yoshida and Ihara, 1993). Thus, PHF-1 provides an especially informative probe with which to test both the feasibility of intracellular antibody delivery and the functional consequences of acutely engaging a defined tau phosphoproteoform in living neuronal cells.

Here, we tested whether DOPC+-based CLD can deliver intact, functional antibodies into living neuronal and neuron-like cells without detectable toxicity. Using PHF-1 together with antibodies directed against other intracellular proteins, we evaluated delivery efficiency, intracellular localization, retention of antigen-binding activity, and consequences for neuronal morphology. We show that CLD rapidly introduces functional full-length antibodies into populations of living cells and primary neurons. Unexpectedly, intracellular engagement of PHF-1-reactive tau was accompanied by rapid neurite retraction and cell body rounding, suggesting that this AD-associated phospho-tau species may also contribute to cell integrity and maintenance of neuronal processes under physiological conditions. These findings establish CLD as a practical approach for acute, antibody-mediated perturbation of intracellular proteoforms and provide a tool for investigating their functions in neuronal development and neurodegenerative disease.

## 2 Methods

### 2.1 Cell lines: maintenance and use

The B103 rat CNS neuroblastoma cell line was a gift of D. Schubert of the Salk Institute, La Jolla, CA. N1E-115 mouse neuroblastoma cells were obtained from American Type Culture Collection (ATCC, Rockville, MD). Neuronal cultures were maintained as described (Zhang et al., 1994) in Dulbecco’s Modified Eagle medium, antibiotic/antimycotic solution, and 10% heat-inactivated fetal calf serum (Mediatech). 3T3 cells (ATCC) were grown in Dulbecco’s Modified Eagle Medium, supplemented with 110 mg/L sodium pyruvate (Mediatech), containing 10% Calf serum (Life Technologies), and 1% antibiotic/antimycotic solution (Mediatech). For experiments, cells were plated on poly-l-lysine coated (200 μg/mL: Sigma) glass coverslips (12 mm or 30 mm) or tissue culture dishes at a density of 200,000–250,000 cells/ml and allowed to grow overnight before use.

### 2.2 Primary retinal cultures

Chick retinal cultures were prepared from embryonic day 8–10 chicks as previously described (Pope et al., 1993). Briefly, retinas were removed and dissociated with 0.1% trypsin in Hank’s balanced salt solution. Trypsinized tissue was resuspended in DMEM media containing 10% heat-inactivated horse serum and 1% antibiotic/antimycotic solution and dissociated cells were plated at a density of 1.0 × 10^6^ cells/ml. Media was changed after 4 h and subsequently as needed.

### 2.3 Probes used

Fyn3 and the Fyn3 peptide antigen were purchased from Santa Cruz Technologies (Santa Cruz, CA). Anti-vinculin and anti-paxillin antibodies were purchased from Transduction Laboratories (Lexington, KY). All antibodies were obtained in a physiological buffer. PHF-1 monoclonal was a gift from Dr. P. Davies (Albert Einstein Medical School, New York, NY). BODIPY®-phallacidin was purchased from Molecular Probes (Eugene, OR).

### 2.4 Cationic liposomal delivery (CLD)

The CLD procedure requires less than 60 min and comprises three steps (Figure 1): (1) The cationic lipid DOPC+ is dried to a film. (2) The film is resuspended in a few microliters of solution containing the probe or protein to be delivered. (3) The lipid-antibody complex is diluted and applied to cultures of living cells, leading to rapid transmembrane delivery of the probe or protein of interest (< 30 min). Cell populations or single cells can then be assayed using desired method.

**Figure 1:**
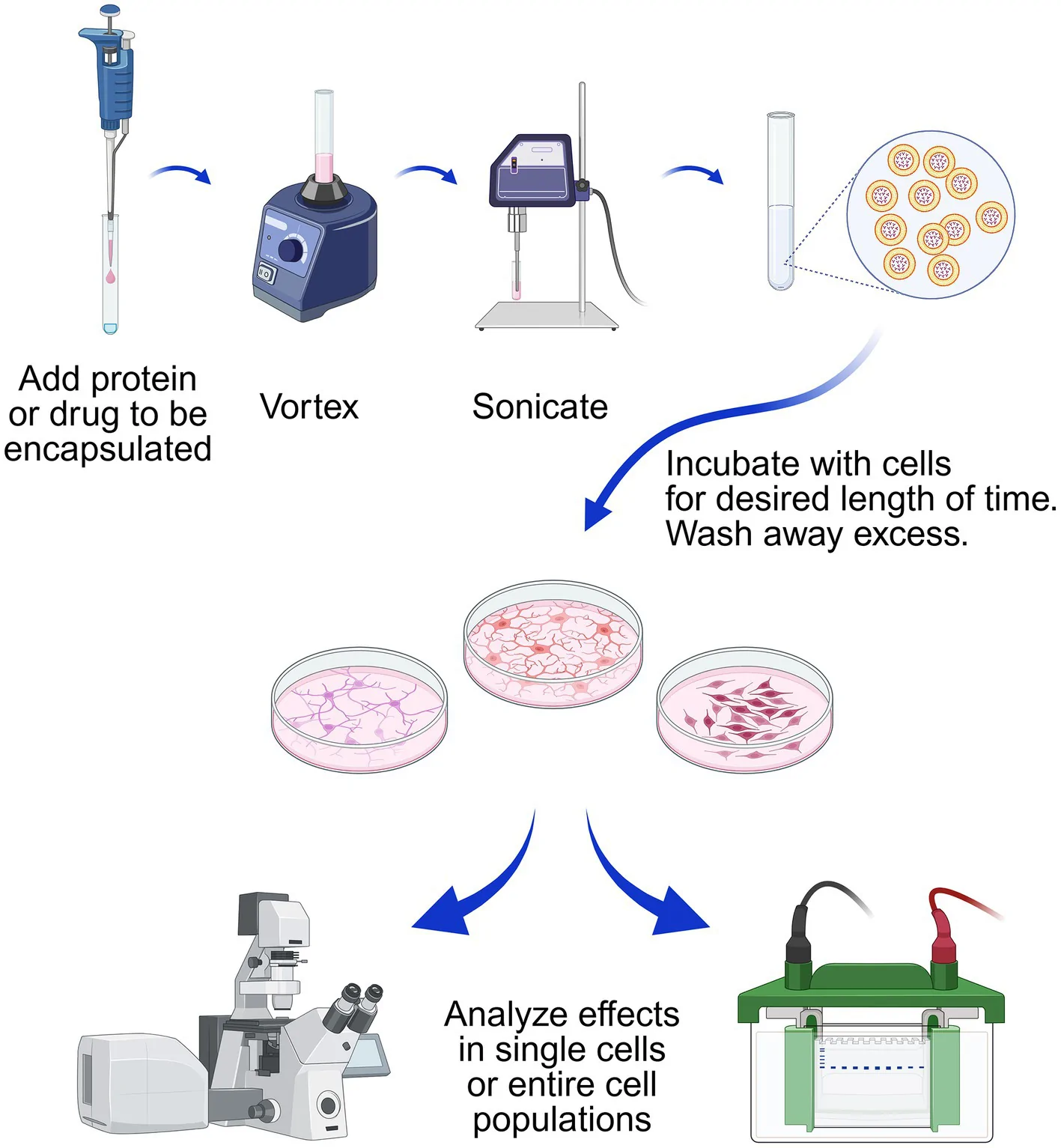
Preparing lipid encapsulated small molecules and antibodies. The “CLD” procedure requires less than 60 min and comprises three steps: (1) the cationic lipid DOPC+ is prepared by evaporating chloroform solvent to give a thin dry film. (2) Antibody, peptide, or probe-lipid complexes are prepared by adding antibody solution to the film, vortexing and sonicating. (3) Antibody is transferred into living cells by incubating them with the complexes, diluted in culture medium, for the desired time. Following the third step, cells are washed with fresh medium to remove excess material and the assay of choice is applied. Created in BioRender. Viola, K. (2026) https://BioRender.com/u2x4d1y.

#### 2.4.1 Lipid preparation

CLD uses a cationic lipid, 1,2-Dioleoyl-sn-Glycero-3-Ethylphosphocholine (DOPC+) (Qiu and MacDonald, 1994; Silinsky et al., 1995), initially developed as an innocuous vehicle for DNA transfection (MacDonald et al., 1999a). DOPC+ is stored in HPLC-grade chloroform to keep it from spontaneously forming liposomes. For typical experiments, 25–100 μg DOPC+ are aliquoted into a clean glass tube, an amount that gives sufficient material for cells in a 35 mm culture. Aliquot(s) are dried under a nitrogen or argon stream, rotating rapidly to distribute the lipid evenly. Samples are placed under vacuum for 30 min. to assure removal of solvent.

#### 2.4.2 Formation of antibody-DOPC+ complexes

Antibody-lipid complex is formed by adding antibody solution to lipid film. A typical volume is 10 μL. Antibodies should be in physiological buffers, free of toxic preservatives. Choice of antibody concentration is empirical; successful results have been obtained with different antibodies at 0.1 mg/mL to 5 mg/mL. After mixing, the suspension is vortexed for 1–2 min, giving a milky suspension of multi-lamellar vesicles (MLVs). Formation of MLVs was initially described by Bangham et al. (1965), Bangham et al. (1974). A clear suspension with white aggregates is undesirable (usually the result of chemically degraded lipid, antibody that is too concentrated, or presence of cryoprotectants such as glycerol). It may be advantageous to convert the MLVs into smaller vesicles; although MLVs are effective at delivering antibody, they can be disruptive to some cells. To make small vesicles, the MLV suspension is sonicated about 30–60 s (e.g., using a cylindrical water-bath sonicator; Special Ultrasonic Cleaner bath sonicator, Model G112SPIT, from Laboratory Supplies Co., Inc.), forming a relatively clear or translucent suspension. Inspection of sonicated antibody-lipid mixtures by atomic force microscopy (not shown) indicated the presence of liposomes with a maximum diameter of 50 nm, a size expected for typical small unilamellar vesicles (Roger et al., 1988; Rols et al., 1998).

#### 2.4.3 CLD of antibody into living cells

Antibody-lipid complexes are diluted into serum-free culture medium before adding to cells. Choice of dilution depends on antibody efficacy and on cell tolerance for DOPC+. (While up to 100 μg DOPC+ /ml culture medium was tolerated by rat hippocampal neurons, chick retinal neurons, and the B103 CNS nerve cell line, it was found that 50 μg DOPC+ in 1 mL culture medium typically was effective at antibody deliver.) Cultures are washed to remove serum before adding antibody-lipid complexes; adaptation to serum-free conditions may be appropriate for some cell types. Incubation for 20–30 min generally is sufficient to provide intracellular antibody, although cells can be incubated with the complexes for over 24 h without causing detectable toxicity. Cells can be washed to remove excess unbound lipid and antibody. Following CLD, specific changes in the physiology of living cells can be evaluated.

### 2.5 Immunocytochemistry

Cultured cells, plated on coverslips as described above, were immunolabeled and processed for microscopy as previously described (Barber et al., 1995; Lambert et al., 1995; Martin et al., 1995; Stevens et al., 1996). Cells were fixed with 3.7% formaldehyde and blocked with 10% normal goat serum in PBS. CLD samples were incubated with a FITC-conjugated anti-mouse or anti-rabbit IgG (1:250, Jackson Immunoresearch Labs, West Grove, PA), or with a biotinylated anti-mouse or anti-rabbit IgG (1:250, Jackson Immunoresearch Labs) followed by a streptavidin-DTAF/Texas Red conjugate (1:250, Jackson Immunoresearch Labs). Traditionally labeled cells were incubated with primary antibody (PHF-1, anti-vinculin, anti-paxillin 1:200; Fyn3 1:100) followed by the appropriate secondary(ies) as above. Coverslips were mounted and analyzed.

### 2.6 Imaging

Epifluorescent and phase/contrast images were collected using a Nikon Diaphot fluorescent microscope with a Hamamatsu CCD digital camera and processed using MetaMorph software (Universal Imaging Corporation, West Chester, PA).

For live-cell imaging, cells were grown on 30 mm glass coverslips coated with poly-L-lysine as described above. Coverslips were then placed in a Dvorak-Stoler chamber (Nicholson Precision Instruments, Gaithersburg, MD) that was then filled with warmed media. Liposomes were introduced to the chamber and allowed to incubate with cells before fresh media was flushed through the chamber to rinse away any unincorporated liposomes. Cells were monitored and imaged using the epifluorescent microscope, either with fluorescence or with phase contrast.

Confocal fluorescent images were collected using a MRC-600 confocal imaging system (Bio-Rad Microscience, England).

Brightfield images were collected using a Leitz Laborlux D microscope (Leitz Wetzlar; Wetzlar, Germany).

## 3 Results

This study primarily uses two neuroblastoma cell lines and primary chick retinal cultures to investigate the possibility that active antibodies can be delivered into living cells without causing membrane disruptions and investigate the impact of the pTau 396/404 antibody, PHF-1, on neuronal and neurite health. B103 is a rat neuroblastoma line derived from the central nervous system that constitutively expresses the pTau 396/404 epitope recognized by the PHF-1 antibody (Lambert et al., 1995). Moreover, these cells have been used in extensive studies involving pTau396/404 (Lambert et al., 1995), aggregated amyloid beta protein (AβOs) (Stevens et al., 1996; Zhang et al., 1996b; Berg et al., 1997), and APP (Jin et al., 1994), with particular attention on neuritogenesis. N1E-115 cells were chosen because they have been used in a variety of tau and neurotransmitter studies (Cohen et al., 1983; Large et al., 1986; Lambert et al., 1995; Oh et al., 2005; Benitez-King et al., 2010; Ivashko-Pachima and Gozes, 2019). Primary chick retinal cultures were used for this study as they were the first primary cells to show the involvement of PHF-1 positive tau in neuronal development (Pope et al., 1993).

### 3.1 CLD mediates the transmembrane delivery of small probes

While microinjection and electroporation are commonly used and effective methods for delivering small probes and proteins into live cells, they have their limitations that make them less suitable for delivering to large populations quickly without causing significant cell loss. Following up on the original reporting of the CLD method in 1996 (Barber et al., 1996) and expanding upon it to determine if CLD is an ideal method for delivering probes with cellular function-modifying capabilities, we wished to test the delivery of several small probes into living cultured cells. Cultured 3T3 fibroblast cells or B103 neuroblastoma cells were incubated with encapsulated small molecules for 20–60 min. Before fixing and imaging to determine distribution. Fluorescent BODIPY-phallacidin could be detected without immunolabeling. To detect the synthetic peptide from the N-terminal domain of the protein tyrosine kinase “Fyn,” cells were fixed and labeled with the Fyn3 antibody and fluorescent secondary before imaging distribution using fluorescent microscopy. Figure 2 shows successful CLD of BODIPY-phallacidin into 3T3 fibroblasts and B103 CNS neuroblastomas. Within 20 min, stress fibers (Figure 2A) and actin in filopodial tips (Figure 2B) were readily seen. CLD also effectively mediated the transmembrane delivery of a synthetic peptide from the N-terminal domain of the protein tyrosine kinase “Fyn” (Figures 2C,D) into B103 cells. After CLD, distribution of the Fyn3 peptide was homogenous in the cytoplasm. Uptake into neuroblastoma cells was imaged using an antibody specific for this sequence. General use of peptides as research tools has been limited, despite efforts to develop “leader” sequences to transfer peptides into cells (Spiller et al., 1998; Okafor et al., 2025). Results here indicate that CLD could supplant leader-sequence peptide technology for most research applications. Dextrans of various sizes (10–70 kDa) also were readily delivered by CLD (data not shown). This may prove valuable in the future as various conjugated dextrans have been developed as fluorescent indicators for ions and small molecules (Molecular Probes, Inc., Eugene, Oregon).

**Figure 2:**
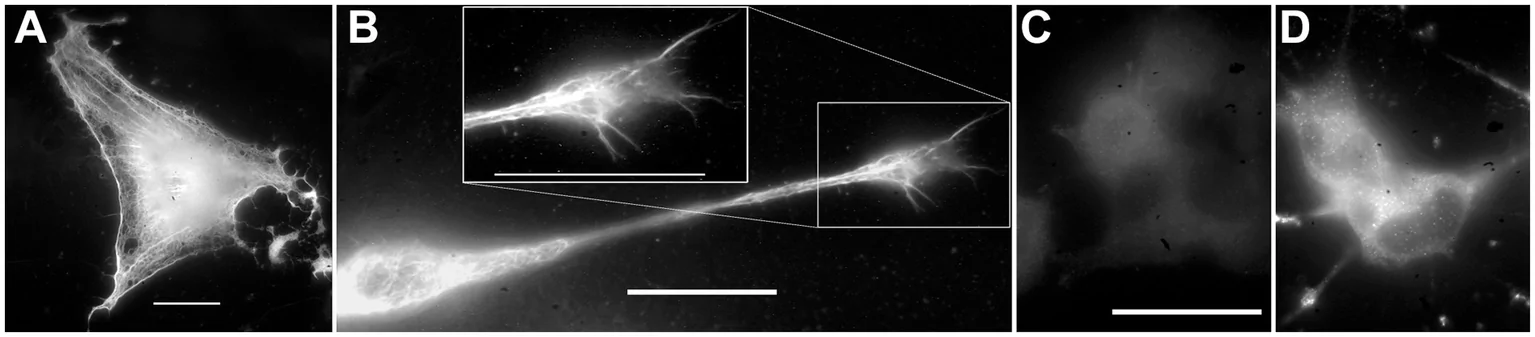
CLD mediates the transmembrane delivery of small probes. CLD was used to deliver small, membrane impermeant probes, phallacidin and Fyn3 antigen peptide, into living cells. **(A,B)** Cells cultured on 30 mm coverslips were placed into a Dvorak-Stoler chamber, incubated for 20 min with BODIPY®-phallacidin:lipid complexes, and visualized live with an epifluorescent microscope. Panel **(A)** shows the actin cytoskeleton of a living 3T3 cell. Panel **(B)** is a B103 rat CNS neuroblastoma cell with the growth cone enlarged in the inset to better visualize the actin cytoskeleton which extends to the tips of the filipodia. **(C,D)** N1E-115 neuroblastoma cells incubated with PBS:lipid controls **(C)** or Fyn3 peptide:lipid complexes **(D)**. Cells were rinsed, fixed, and immunolabeled with Fyn3 antibody. Low level staining in controls **(C)** is consistent with minimal endogenous Fyn expression in N1E-115 cells. Panel **(D)** shows the distribution of the Fyn3 peptide throughout the cells after delivery via CLD. Scale bars = 25 μm.

### 3.2 CLD effectively delivers antibody into an entire population of living cells

To further expand upon this method and its potential usefulness at distributing functional proteins to entire populations of living cultured cells, delivery of antibodies was next investigated. Delivery of small molecules, peptides, and other small probes is very useful, but rapid delivery of larger proteins like antibodies to entire populations would significantly improve the ability to investigate or modify cellular processes *in vitro*. Cultured N1E-115 cells were incubated with either encapsulated Fyn3 antibody, empty liposomes and Fyn3 antibody (unencapsulated), or nothing for 1 h. Cells were then rinsed with fresh media and fixed. Cells incubated with nothing were immunolabeled using traditional immunocytochemistry methods, while the cells incubated with liposomes were probed with biotinylated secondary only. Cells were then treated with Streptavidin-DTAF to visualize and imaged with a Leitz brightfield microscope to allow analysis of larger populations at a time. Results show that Fyn3 antibody delivered into N1E-115 cells using CLD (Figure 3B) showed nearly identical labeling of the population as that seen in cultures that were fixed and immunolabeled using traditional protocols (Figure 3A). The cells treated with a mixture of empty liposomes and unencapsulated Fyn3 antibody showed no labeling (Figure 3C). These results demonstrate that CLD can deliver larger proteins into living cells within 60 min. These results also demonstrate that the presence of liposomes is not sufficient to facilitate antibody uptake. The antibody must be encapsulated in the liposome to be delivered into the cells.

**Figure 3:**
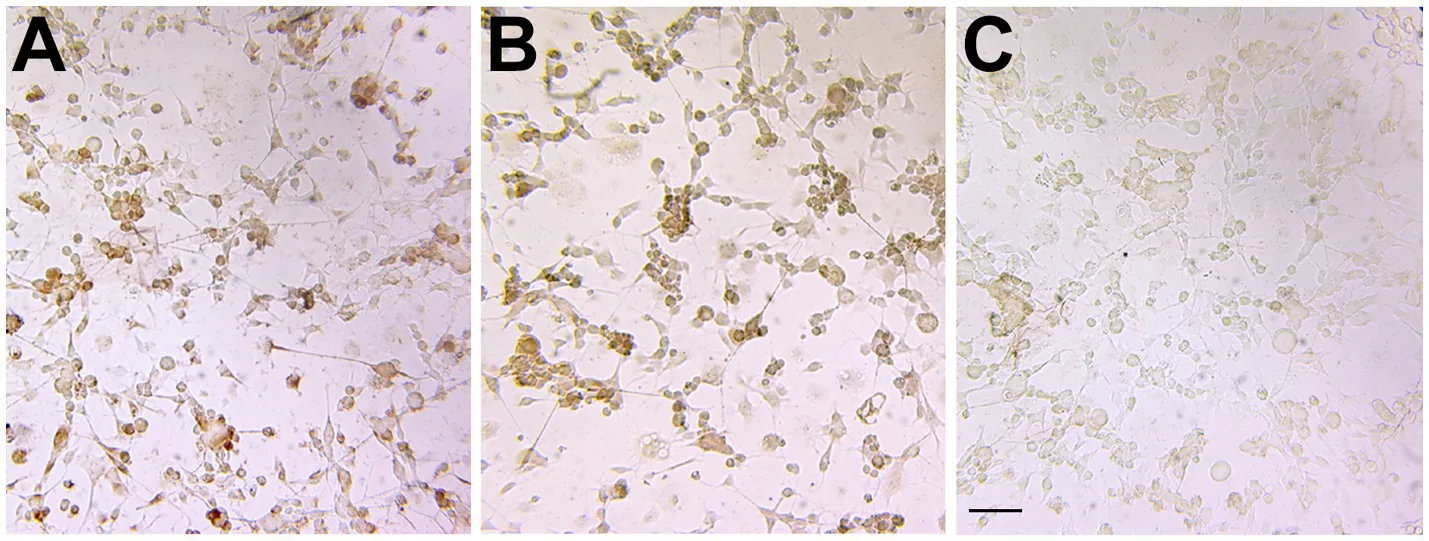
CLD effectively delivers antibody into an entire population of living cells. **(A)** Fyn3 antibody was delivered via CLD into N1E-115 cells for 60 min. Cells were then fixed and probed with secondary antibody followed by DAB visualization. Controls consisted of cells that were fixed prior to labeling with Fyn3 antibody and secondary **(B)** or cells that were incubated with PBS-filled liposomes and unencapsulated Fyn3 antibody **(C)**. All images were captured with the Leitz Laborlux D brightfield microscope. Panel **(A)** shows remarkably similar labeling of Fyn3 as compared to cultures that were fixed and immunolabeled using traditional immunofluorescence protocols **(B)**. Panel **(C)** shows that the signal detected is dependent upon the uptake and binding of the Fyn3 antibody. Scale bar = 50 μm.

### 3.3 Antibodies delivered into living cells actively target their antigen of interest

CLD delivery of probes and antibodies into living cells is only a relevant procedure if the probes and antibodies remain active, targeting the protein or process of interest just as they would if they were delivered through alternative means. These experiments were designed to demonstrate that CLD delivers functional antibodies into living cells, as visualized by confocal immunofluorescence. Cultured B103 cells were incubated with liposome encapsulated antibodies against paxillin (Figures 4A,B) or vinculin (Figures 4C,D) for 30 min, washed with clean media and fixed. Cells were then labeled with appropriate fluorescently-conjugated secondary antibodies and imaged with an inverted epifluorescent microscope. Results show paxillin and vinculin antibodies were delivered into B103 cells, where they labeled cells as predicted, including at putative focal contacts in the tips of growth cones. The data shown in Figure 4 shows representative images of vinculin and paxillin detection after CLD, two proteins found in focal contacts. Focal contacts, which occur in neurons as well as other cells (Zhang et al., 1994; Grant et al., 1995; Stevens et al., 1996; Zhang et al., 1996a, b; Berg et al., 1997; Wojciak-Stothard et al., 1997), comprise protein complexes near the plasma membrane. Their discrete loci provide a validating assay for functional intracellular antibodies.

**Figure 4:**
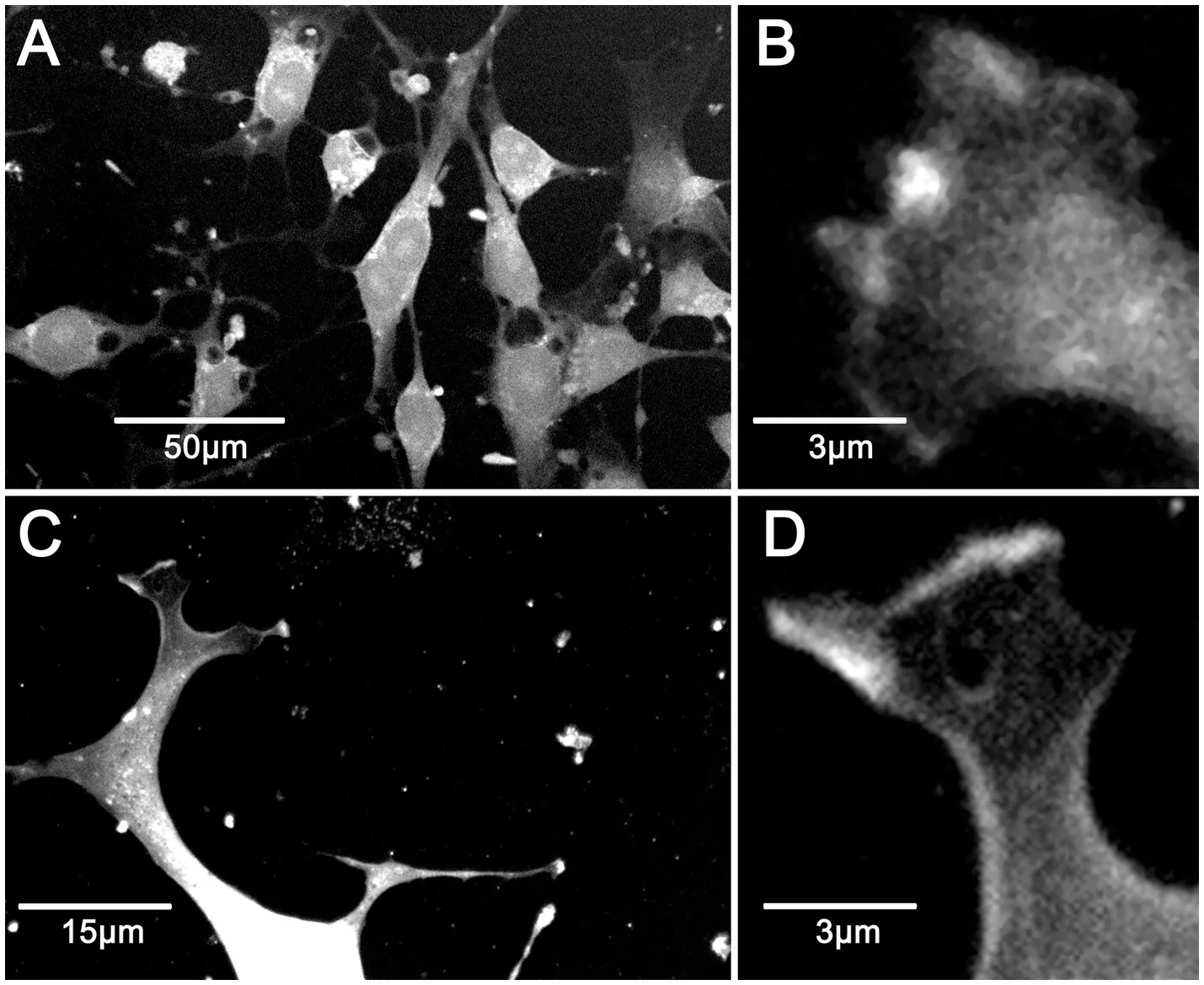
Antibodies delivered into living cells remain active. CLD was used to deliver antibodies against focal contact proteins into B103 cells. After 30 min incubation, cells were rinsed with media, fixed, and antibodies immunolocalized with fluorescent-conjugated secondaries. Cells were imaged with the Bio-Rad MRC-600 confocal imaging system. **(A,B)** CLD of anti-paxillin antibody. **(C,D)** CLD of anti-vinculin antibody. Both antibodies show staining of cell bodies, extending to the growth cones and labeling putative focal contacts.

### 3.4 PHF-1 antibody delivery into NE1-115 cells is similarly efficient at labeling an entire cell population

Ultimately, the goal of this project is to deliver proteoform-specific functional antibodies into living cells in a manner that enables the study of cellular processes and molecular mechanisms involved in Alzheimer’s disease. PHF-1 was developed as a diagnostic for phospho-tau in Alzheimer’s disease (Bodenteich et al., 1992), but it recognizes the same epitope found in neuroblastomas (Pope et al., 1993; Lambert et al., 1994) and transiently in developing axons (Pope et al., 1994). Cultured N1E-115 neuronal cells were either fixed and labeled using traditional immunofluorescence techniques (Figure 5A), incubated live with PBS filled liposomes and PHF1 antibody added to the media for 1 h (Figure 5B), or incubated live with PHF1 filled liposomes for 1 h (Figure 5C). Data show that delivery into cells is dependent upon the antibody being encapsulated in the liposomes and that the distribution within the cells and within the population after liposome delivery is nearly identical to that seen when immunolabeling fixed cells. Delivery of PHF1 antibody against phosphorylated tau shows a strikingly similar distribution within the population and within the cells.

**Figure 5:**
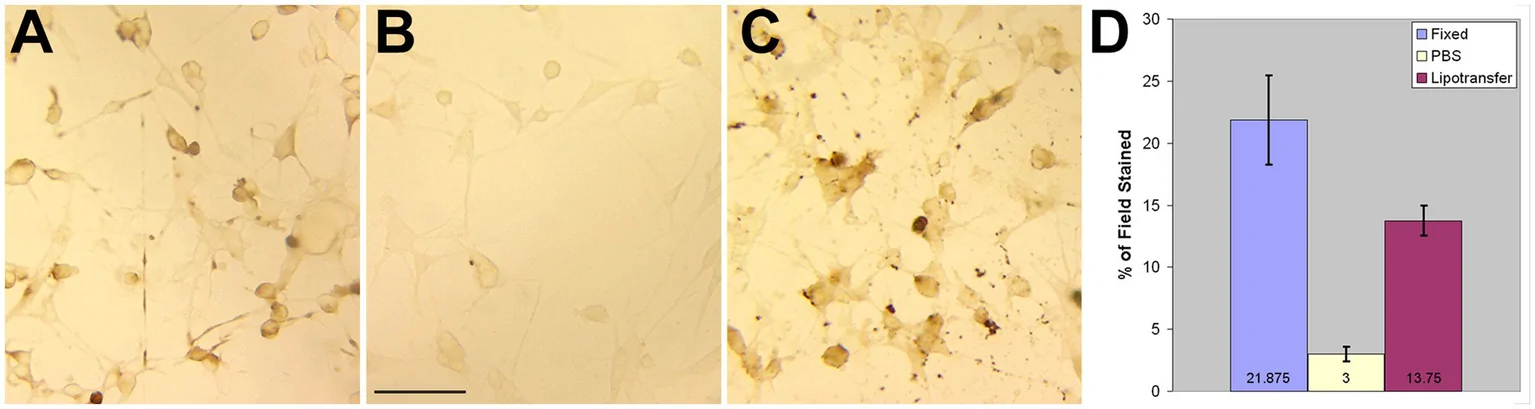
PHF-1 antibody delivery into NE1-115 cells is similarly efficient at labeling an entire cell population. Delivery of PHF1 antibody against phosphorylated tau shows a strikingly similar distribution within the population and within the cells. Cultured N1E-115 neuronal cells were either **(A)** fixed and labeled using traditional immunofluorescence techniques, **(B)** incubated live with PBS filled liposomes and PHF1 antibody added to the media for 1 h, or **(C)** incubated live with PHF1 filled liposomes for 1 h. All cells were then washed, fixed, incubated with secondary antibody, and visualized with DAB. Brightfield images were then captured with the Leitz microscope. Data show that delivery into cells is dependent upon the antibody being encapsulated in the liposomes. Data also show that the distribution within the cells and within the population after liposome delivery is nearly identical to that seen when immunolabeling fixed cells. **(D)** Analysis of the percentage of the population that was labeled showed that CLD delivered antibody into approximately 65% of the population that was labeled using traditional immunolabeling methods (*n* = 9+, the number of fields captured per condition across 4 independent experiments). Scale bar = 50 μm.

### 3.5 PHF-1 antibody delivery into a neuronal cell line shows proteoform-specific fibrillar distribution

To further demonstrate the intracellular delivery of a functional proteoform-specific antibody into a population of living neurons, we again used PHF-1 antibody and cultured central nervous system (CNS) neurons. Cultured B103 CNS neurons were incubated with encapsulated PHF-1 antibody for 30 min, washed and fixed. As a control, cultured B103 cells were fixed and immunolabeled with PHF-1 antibody. Both conditions were treated with fluorescent secondary antibody before imaging with an epifluorescence microscope. Delivery of PHF-1 into live B103 CNS neuroblastoma cells gave filamentous patterns of immunoreactivity (Figure 6B) predicted by traditional labeling after fixation and permeabilization with acetone or 0.01% Triton X-100 (Figure 6A). Detection of the PHF-1-positive fibrillar arrays (Figure 6) demonstrate that the putative focal contacts imaged in Figure 4 are not methodological artifacts. The data verify the successful delivery of active proteoform-specific antibodies into living cells.

**Figure 6:**
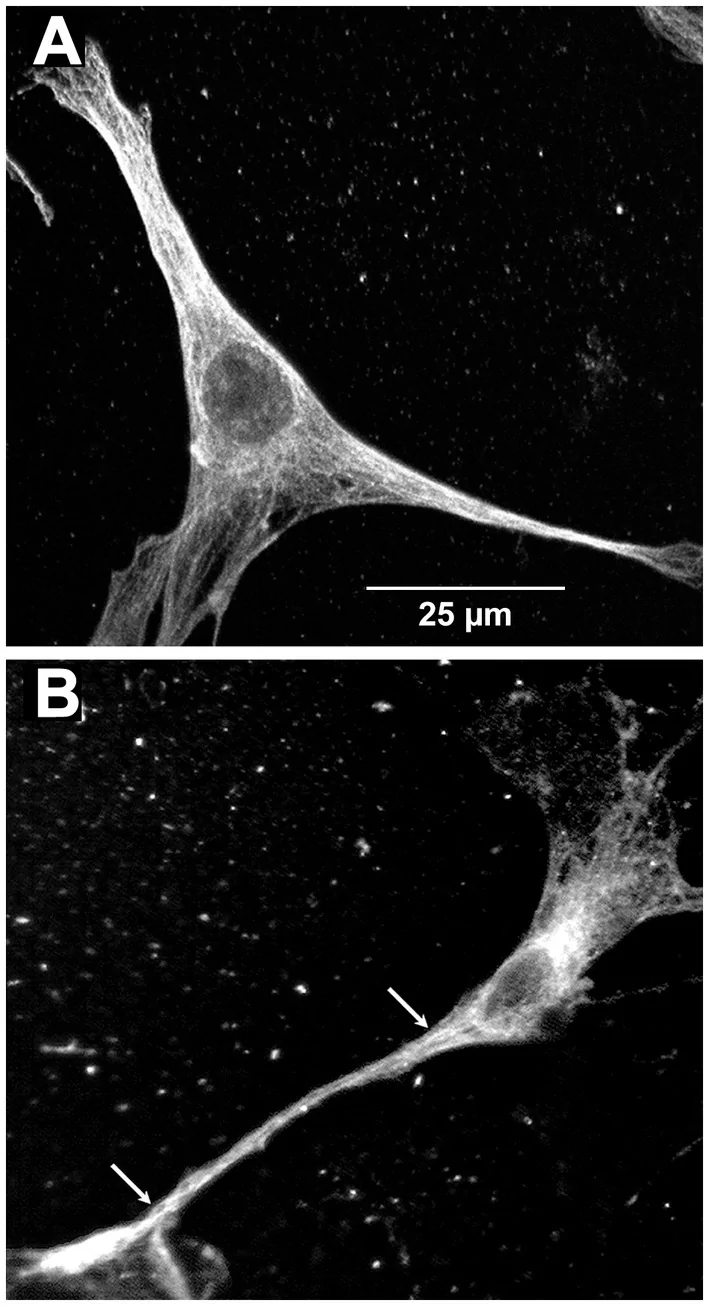
PHF-1 antibody delivery into a neuronal cell line shows proteoform-specific fibrillar distribution. PHF-1 monoclonal antibody, which recognizes a phosphorylated epitope of tau, localizes to the fibrillar microtubule array in B103 cells (Lambert et al., 1995). CLD of PHF-1 antibody into B103 cells followed by fluorescence localization showed a similar immunofluorescence pattern. An image of PHF-1 antibody distribution in pre-fixed cells, processed by standard immunofluorescence methods **(A)** shows a fibrillar distribution of the antibody in the cytoplasm of the cell. PHF-1 delivered by CLD **(B)** into a living cell shows a similar distribution.

### 3.6 Delivery into cultured primary CNS neurons is independent of cell proliferation-state

CLD efficiently delivered antibodies to entire populations of neuronal cell lines, but it is essential to also effectively transfer antibody into post-mitotic neurons. Using cultured primary chick retinal cultures (White Leghorn, E8 retina), neuronal populations were treated with liposome encapsulated PHF-1 antibody for 30 min, then fixed and treated with secondary antibody to visualize. Figure 7 shows PHF-1 distribution after delivery into primary cultures from embryonic chick retinas. Virtually all cells took up antibody (Figure 7A) while cells treated with PBS filled liposomes showed no labeling (Figure 7B). At higher magnification (Figure 7C) and fluorescent confocal imaging (Figure 7D), PHF-1 imaged within retina neurons after delivery shows a distribution observed previously in fixed, traditionally immunolabeled cells (Pope et al., 1993; Pope et al., 1994). Taken together, data demonstrate the efficient transfer of antibody into living neurons as well as transformed cell lines in a manner that retains function and permits diffusion through the cytoplasm. Since separate addition of antibody and empty liposomes to cultures resulted in little or no intracellular antibody, it appears that the concentrated antibody-lipid complex is the critical delivery material.

**Figure 7:**
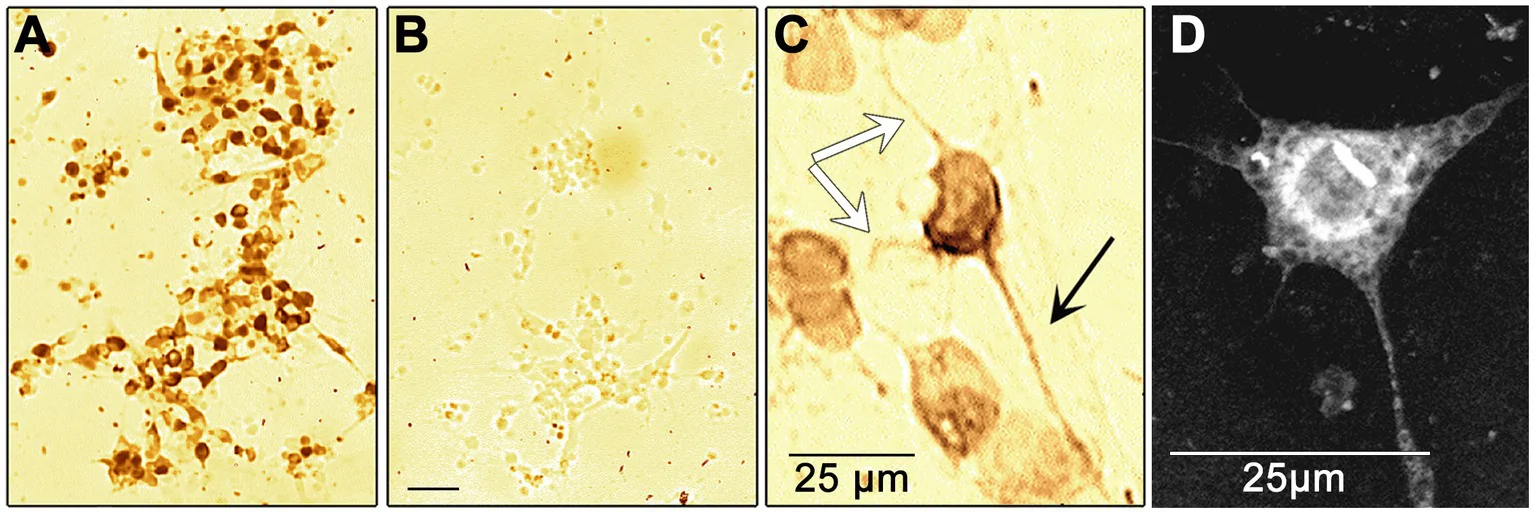
Delivery into cultured primary CNS neurons is independent of cell proliferation-state. The efficacy of CLD for post-mitotic neurons is demonstrated by delivery of PHF-1 into neurons cultured from embryonic chick retinas. A single batch-wise delivery of PHF-1 **(A)** labeled essentially all cells. Controls with PBS:lipid **(B)** show background for comparison. Even though all cells receive antibody, some cells stain more intensely than others **(A,C)**. This result is explained by previous findings that only ganglion neurons contain antigen, which is discretely localized and thus gives highly concentrated signal (Pope et al., 1993). At higher magnifications **(C,D)**, PHF-1 imaged within retinal neurons after CLD shows the same distribution observed previously in fixed cells (Pope et al., 1993; Pope et al., 1994). Panel **(C)** shows a polarized PHF-1 distribution, with one process containing greater label (dark arrow) than the others (open arrows). Panel **(D)** shows a dense circumnuclear PHF-1 pattern caputured using the confocal microscope. Both patterns recapitulate observations made by traditional methods (Pope et al., 1993). Scale bar = 50 μm in panel **(B)**.

### 3.7 Intracellular PHF-1 antibody engagement is associated with rapid neurite retraction

PHF-1 antibody was developed against an Alzheimer’s disease-associated phospho-tau epitope that has also been shown to be present during neuronal development (Greenberg, 1990; Pope et al., 1993; Busciglio et al., 1995; Hefti et al., 2019). To investigate the consequences of intracellular engagement of the pTau Ser396/404 tau pool, B103 cultures were exposed to CLD-delivered PHF-1 and monitored by live imaging. Within approximately three hours, cells receiving active PHF-1 exhibited marked neurite retraction and cell body rounding (Figure 8A) relative to cultures exposed to heat-inactivated PHF-1 (Figure 8B) or PBS-loaded liposomes (not shown). These findings demonstrate that intracellular delivery of a functional PHF-1 antibody is sufficient to produce rapid alterations in neuronal morphology. While consistent with a role for PHF-1-reactive tau species in maintaining neurite architecture, the present experiments do not establish the underlying mechanism. Antibody binding may interfere with physiological functions of phosphorylated tau through multiple non-mutually-exclusive mechanisms (Wang and Mandelkow, 2016; Mueller et al., 2021; Parra Bravo et al., 2024). These data demonstrate that intracellular engagement of the PHF-1 epitope perturbs neuronal process stability, but does not suggest that the pTau Ser396/404 modification itself is uniquely required for neurite maintenance.

**Figure 8:**
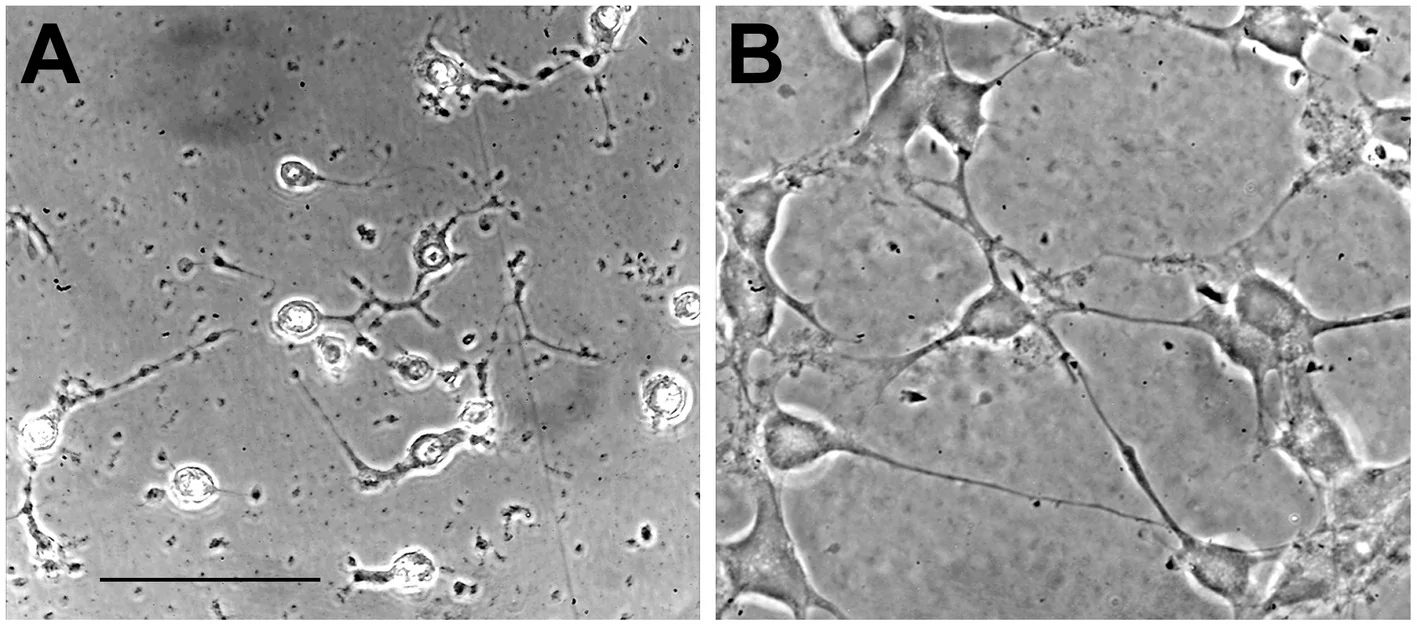
Intracellular PHF-1 antibody engagement induces rapid neurite retraction. B103 cells were cultured on 30 mm coverslips and monitored by live imaging. **(A)** Active PHF-1 delivered via CLD produced neurite retraction and cell body rounding within three hours. **(B)** Heat-inactivated PHF-1 had no detectable effect on cell morphology, even after prolonged incubation. These findings demonstrate that CLD delivers functionally active antibodies into living cells and that intracellular engagement of PHF-1-reactive tau species is associated with rapid changes in neurite morphology. Data further show that the lipid vehicle is not visibly disruptive. Scale bar = 50 μm.

Taken together, these data show the feasibility of using CLD with DOPC+ lipid as the carrier to deliver cargo, including full-length antibodies, intracellularly to entire populations of live cells without inducing visible signs of neurotoxicity. These populations of cells can then be used in a variety of biochemical, molecular, or microscopy assays to evaluate the impact of the perturbances.

## 4 Discussion

The ability to selectively probe endogenous protein proteoforms in living neurons remains a major challenge in neuroscience and neurodegeneration research. Although genetic manipulation, recombinant expression, and intracellular antibody expression systems have provided valuable insights into protein function, these approaches typically require extended experimental timelines and often affect all forms of a target protein simultaneously (Gallardo et al., 2019; Goodwin et al., 2021; Rodrigues Martins et al., 2023). Here, we demonstrate that cationic liposomal delivery (CLD) efficiently introduces functional full-length antibodies into living neuronal cell lines and primary neurons without detectable toxicity. Importantly, delivered antibodies retained intracellular target recognition and generated staining patterns consistent with the known localization of endogenous proteins including tau, vinculin, paxillin, and Fyn. These findings establish CLD as a practical approach for acute intracellular antibody delivery and provide a means for probing endogenous proteoforms in their native cellular environment.

A key advantage of CLD is its ability to achieve rapid intracellular access without requiring viral vectors, genetic modification, recombinant protein engineering, or prolonged expression periods. This capability is particularly relevant for proteins such as tau, which exist as complex populations of proteoforms generated through extensive post-translational modification. Increasing evidence suggests that individual tau proteoforms may possess distinct biological properties and pathological relevance, yet tools capable of selectively probing specific phospho-tau species in living neurons remain limited (Smith et al., 2013; Aebersold et al., 2018; Joly et al., 2026). By enabling intracellular delivery of well-characterized monoclonal antibodies, CLD provides a strategy for acutely manipulating specific proteoforms while preserving the endogenous cellular context.

The most significant biological finding of this study emerged from the intracellular delivery of PHF-1, an antibody that recognizes tau phosphorylated at Ser396 and Ser404 (Greenberg, 1990; Otvos Jr et al., 1994). Following PHF-1 delivery, neurons exhibited rapid neurite retraction and cell-body rounding, with clear morphological alterations developing within a few hours. Notably, these effects were not observed following treatment with heat-inactivated PHF-1, vehicle controls, or other intracellular antibodies. The specificity of this response argues against a nonspecific consequence of liposomal delivery or intracellular antibody accumulation and instead supports the conclusion that intracellular engagement of PHF-1-reactive tau species perturbs processes involved in maintaining normal neuronal morphology.

The mechanism underlying this response remains to be established. One possibility is that PHF-1 binding interferes with physiological interactions between phosphorylated tau and components of the cytoskeleton that contribute to neurite stability. Alternatively, antibody binding may alter the conformation, trafficking, molecular interactions, or subcellular organization of the PHF-1-reactive tau population. Because antibodies are multivalent molecules, intracellular crosslinking of tau species could also contribute to the observed phenotype. Although the present experiments do not distinguish among these possibilities, the rapid onset of neurite retraction suggests PHF-1-reactive tau is involved in dynamic cellular processes supporting neuronal architecture. Future studies examining cytoskeletal organization, microtubule stability, and tau-binding partners following PHF-1 delivery may help elucidate the underlying mechanism.

These observations are particularly noteworthy given the long-standing association of the PHF-1 epitope with Alzheimer’s disease pathology. Phosphorylation of tau at Ser396 and Ser404 is a prominent feature of paired helical filaments and neurofibrillary tangles and is commonly regarded as a marker of pathological tau accumulation (Greenberg, 1990; Otvos Jr et al., 1994; Hefti et al., 2019). However, several studies have also reported PHF-1 immunoreactivity in the developing nervous system, indicating that this phospho-tau species is not restricted to disease states (Brion et al., 1993; Kenessey and Yen, 1993b; Pope et al., 1993). The coexistence of PHF-1 reactivity in both developmental and pathological contexts has raised the possibility that this modified form of tau serves physiological functions that may become dysregulated during disease progression. The present findings are consistent with this interpretation and suggest that PHF-1-reactive tau species contribute to the maintenance of neurite integrity under normal conditions.

Importantly, the data should not be interpreted as demonstrating that phosphorylation at Ser396/404 is itself required for neurite maintenance. Rather, the experiments show that acute intracellular engagement of the PHF-1-reactive tau population is sufficient to induce rapid structural changes in neurons. Since PHF-1 recognizes a specific phospho-dependent conformational state within a broader pool of tau molecules, the observed phenotype most directly implicates PHF-1-reactive tau species rather than any individual phosphorylation event. Additional studies employing complementary phospho-specific reagents and biochemical approaches will be necessary to determine the precise molecular determinants responsible for the observed effects.

Beyond the specific findings related to tau biology, this work highlights the broader utility of intracellular antibody delivery as a functional discovery platform. In the present study, PHF-1 was initially employed as a model cargo to assess intracellular delivery. Unexpectedly, its introduction revealed a robust and previously unappreciated neuronal phenotype. This outcome illustrates how intracellular antibodies can function not only as imaging and detection reagents but also as acute perturbation tools for dissecting the functions of endogenous proteoforms. Similar strategies could be applied to investigate disease-associated protein species generated by phosphorylation, truncation, acetylation, ubiquitination, and other post-translational modifications across a wide range of neurodegenerative disorders.

There are limitations that should be considered. The present experiments do not identify the molecular pathway responsible for PHF-1-induced neurite retraction, nor do they determine whether the observed effects are reversible. In addition, the current findings were obtained in cultured neuronal systems, and future studies will be needed to determine whether similar responses occur in intact neural circuits and *in vivo*. Defining concentration dependence, evaluating additional phospho-tau antibodies, and assessing downstream cytoskeletal changes will further strengthen understanding of the biological significance of PHF-1-reactive tau species.

In conclusion, the present study establishes CLD as an effective and experimentally accessible method for delivering functional full-length antibodies into living neuronal populations. More importantly, application of this approach revealed that acute intracellular engagement of PHF-1-reactive tau species rapidly destabilizes neurites and alters neuronal morphology. These findings support a functional role for this phospho-tau population in maintaining neuronal architecture and demonstrate the value of intracellular antibody delivery as a strategy for probing the biological activities of endogenous proteoforms in living neurons.

## Data Availability

The raw data supporting the conclusions of this article will be made available by the authors, without undue reservation.

## References

[ref1] Aebersold R. Agar J. N. Amster I. J. Baker M. S. Bertozzi C. R. Boja E. S. . (2018). How many human proteoforms are there? Nat. Chem. Biol. 14, 206–214. doi: 10.1038/nchembio.2576 PMC5837046 29443976

[ref2] Ashton N. J. Leuzy A. Karikari T. K. Mattsson-Carlgren N. Dodich A. Boccardi M. . (2021). The validation status of blood biomarkers of amyloid and phospho-tau assessed with the 5-phase development framework for AD biomarkers. Eur. J. Nucl. Med. Mol. Imaging 48, 2140–2156. doi: 10.1007/s00259-021-05253-y, 33677733 PMC8175325

[ref3] Augustinack J. C. Schneider A. Mandelkow E. M. Hyman B. T. (2002). Specific tau phosphorylation sites correlate with severity of neuronal cytopathology in Alzheimer's disease. Acta Neuropathol. 103, 26–35. doi: 10.1007/s004010100423, 11837744

[ref4] Bangham A. D. Hill M. W. Miller N. G. A. (1974). “Preparation and use of liposomes as models of biological membranes,” in Methods in Membrane Biology: Volume 1, ed. Korn E. D. (Boston, MA: Springer US), 1–68.

[ref5] Bangham A. D. Standish M. M. Watkins J. C. (1965). Diffusion of univalent ions across the lamellae of swollen phospholipids. J. Mol. Biol. 13, 238–252. doi: 10.1016/s0022-2836(65)80093-6, 5859039

[ref6] Barber K. Enam S. A. Bodovitz S. Falduto M. Frail D. Klein W. L. (1995). Particulate forms of APP in the extracellular milieu of cultured cells. Exp. Neurol. 132, 42–53. 10.1016/0014-4886(95)90057-8 7720825

[ref7] Barber K. Mala R. R. Lambert M. P. Qiu R. MacDonald R. C. Klein W. L. (1996). Delivery of membrane-impermeant fluorescent probes into living neural cell populations by lipotransfer. Neurosci. Lett. 207, 17–20. doi: 10.1016/0304-3940(96)12497-6, 8710199

[ref8] Barthelemy N. R. Li Y. Joseph-Mathurin N. Gordon B. A. Hassenstab J. Benzinger T. L. S. . (2020). A soluble phosphorylated tau signature links tau, amyloid and the evolution of stages of dominantly inherited Alzheimer's disease. Nat. Med. 26, 398–407. doi: 10.1038/s41591-020-0781-z PMC7309367 32161412

[ref9] Benitez-King G. Ortiz-Lopez L. Jimenez-Rubio G. Ramirez-Rodriguez G. (2010). Haloperidol causes cytoskeletal collapse in N1E-115 cells through tau hyperphosphorylation induced by oxidative stress: implications for neurodevelopment. Eur. J. Pharmacol. 644, 24–31. doi: 10.1016/j.ejphar.2010.06.057 20621083

[ref10] Berg M. M. Krafft G. A. Klein W. L. (1997). Rapid impact of beta-amyloid on paxillin in a neural cell line. J. Neurosci. Res. 50, 979–989. doi: 10.1002/(SICI)1097-4547(19971215)50:6<979::AID-JNR8>3.0.CO;2-A, 9452012

[ref11] Bodenteich A. Mitchell L. G. Merril C. R. Miller B. E. Ghanbari H. A. Heegaard N. H. (1992). Immunochemical characterization of a monoclonal antibody specific for Alzheimer's disease associated protein. J. Neuroimmunol. 41, 111–115. doi: 10.1016/0165-5728(92)90202-v, 1460088

[ref12] Braak H. Braak E. (1991). Neuropathological stageing of Alzheimer-related changes. Acta Neuropathol. 82, 239–259. doi: 10.1007/BF00308809, 1759558

[ref13] Brion J. P. Smith C. Couck A. M. Gallo J. M. Anderton B. H. (1993). Developmental changes in tau phosphorylation: fetal tau is transiently phosphorylated in a manner similar to paired helical filament-tau characteristic of Alzheimer's disease. J. Neurochem. 61, 2071–2080. doi: 10.1111/j.1471-4159.1993.tb07444.x, 8245963

[ref14] Busciglio J. Lorenzo A. Yeh J. Yankner B. A. (1995). beta-amyloid fibrils induce tau phosphorylation and loss of microtubule binding. Neuron 14, 879–888. doi: 10.1016/0896-6273(95)90232-5, 7718249

[ref15] Chan A. Tsourkas A. (2024). Intracellular protein delivery: approaches, challenges, and clinical applications. BME Front. 5:0035. doi: 10.34133/bmef.0035, 38282957 PMC10809898

[ref16] Chen Y. Yu Y. (2023). Tau and neuroinflammation in Alzheimer's disease: interplay mechanisms and clinical translation. J. Neuroinflammation 20:165. doi: 10.1186/s12974-023-02853-3, 37452321 PMC10349496

[ref17] Cohen N. M. Schmidt D. M. McGlennen R. C. Klein W. L. (1983). Receptor-mediated increases in phosphatidylinositol turnover in neuron-like cell lines. J. Neurochem. 40, 547–554. doi: 10.1111/j.1471-4159.1983.tb11317.x, 6822837

[ref18] Fang F. Xu T. Chien Hagar H. T. Hovde S. Kuo M. H. Sun L. (2024). Pilot study for deciphering post-translational modifications and Proteoforms of tau protein by capillary electrophoresis-mass spectrometry. J. Proteome Res. 23, 5085–5095. doi: 10.1021/acs.jproteome.4c00587, 39327902 PMC11536466

[ref19] Fulcher J. M. Ives A. N. Tasaki S. Kelly S. S. Williams S. M. Fillmore T. L. . (2025). Discovery of Proteoforms associated with Alzheimer's disease through quantitative top-down proteomics. Mol. Cell. Proteomics 24:100983. doi: 10.1016/j.mcpro.2025.100983, 40334744 PMC12173667

[ref20] Gallardo G. Wong C. H. Ricardez S. M. Mann C. N. Lin K. H. Leyns C. E. G. . (2019). Targeting tauopathy with engineered tau-degrading intrabodies. Mol. Neurodegener. 14:38. doi: 10.1186/s13024-019-0340-6, 31640765 PMC6805661

[ref21] Goedert M. Crowther R. A. Scheres S. H. W. Spillantini M. G. (2024). Tau and neurodegeneration. Cytoskeleton 81, 95–102. doi: 10.1002/cm.21812, 38073060 PMC7615684

[ref22] Goedert M. Jakes R. Crowther R. Six J. Lübke U. Vandermeeren M. . (1993). The abnormal phosphorylation of tau protein at Ser-202 in Alzheimer disease recapitulates phosphorylation during development. Proc. Natl. Acad. Sci. USA 90, 5066–5070. doi: 10.1073/pnas.90.11.5066, 8506352 PMC46655

[ref23] Goedert M. Spillantini M. G. (2011). Pathogenesis of the tauopathies. J. Mol. Neurosci. 45, 425–431. doi: 10.1007/s12031-011-9593-4 21785996

[ref24] Goodwin M. S. Sinyavskaya O. Burg F. O’Neal V. Ceballos-Diaz C. Cruz P. E. . (2021). Anti-tau scFvs targeted to the cytoplasm or secretory pathway variably modify pathology and neurodegenerative phenotypes. Mol. Ther. 29, 859–872. doi: 10.1016/j.ymthe.2020.10.007, 33128896 PMC7854277

[ref25] Grant S. G. Karl K. A. Kiebler M. A. Kandel E. R. (1995). Focal adhesion kinase in the brain: novel subcellular localization and specific regulation by Fyn tyrosine kinase in mutant mice. Genes Dev. 9, 1909–1921. doi: 10.1101/gad.9.15.1909 7544314

[ref26] Greenberg S. G. (1990). The developmental regulation of PHF-1 reactive tau proteins. Neurobiol. Aging 11:285.

[ref27] Hefti M. M. Kim S. Bell A. J. Betters R. K. Fiock K. L. Iida M. A. . (2019). Tau phosphorylation and aggregation in the developing human brain. J. Neuropathol. Exp. Neurol. 78, 930–938. doi: 10.1093/jnen/nlz073, 31504677 PMC6751069

[ref28] Henkins K. M. Sokolow S. Miller C. A. Vinters H. V. Poon W. W. Cornwell L. B. . (2012). Extensive p-tau pathology and SDS-stable p-tau oligomers in Alzheimer's cortical synapses. Brain Pathol. 22, 826–833. doi: 10.1111/j.1750-3639.2012.00598.x, 22486774 PMC3410970

[ref29] Irwin D. J. Cohen T. J. Grossman M. Arnold S. E. Xie S. X. Lee V. M. . (2012). Acetylated tau, a novel pathological signature in Alzheimer's disease and other tauopathies. Brain 135, 807–818. doi: 10.1093/brain/aws013 PMC3286338 22366796

[ref30] Ivashko-Pachima Y. Gozes I. (2019). A novel microtubule-tau association enhancer and neuroprotective drug candidate: ac-SKIP. Front. Cell. Neurosci. 13:435. doi: 10.3389/fncel.2019.00435, 31632241 PMC6779860

[ref31] Jin L. W. Ninomiya H. Roch J. M. Schubert D. Masliah E. Otero D. A. . (1994). Peptides containing the RERMS sequence of amyloid beta/A4 protein precursor bind cell surface and promote neurite extension. J. Neurosci. 14, 5461–5470. doi: 10.1523/JNEUROSCI.14-09-05461.1994, 8083748 PMC6577069

[ref32] Joly J. Budamagunta V. Zhang Z. Nortman B. Jouzi M. Bhatnagar R. . (2026). Large-scale single-molecule analysis of tau proteoforms. Nat. Methods 23, 1786–1797. doi: 10.1038/s41592-026-03188-6, 42697987 PMC13545013

[ref33] Kenessey A. Yen S.-H. C. (1993). The extent of phosphorylation of fetal tau is comparable to that of PHF-tau from Alzheimer paired helical filaments. Brain Res. 629, 40–46. doi: 10.1016/0006-8993(93)90478-6, 8287279

[ref34] Kosik K. S. Orecchio L. D. Bakalis S. Neve R. L. (1989). Developmentally regulated expression of specific tau sequences. Neuron 2, 1389–1397. doi: 10.1016/0896-6273(89)90077-9, 2560640

[ref35] Kumar M. Schlaffner C. N. Tang S. Beuvink M. A. Viode A. Mair W. . (2026). Molecular features of human pathological tau distinguish tauopathy-associated dementias. Cell 189, 956–968.e13. doi: 10.1016/j.cell.2025.12.036, 41616780 PMC13075643

[ref36] Lambert M. P. Sabo S. Zhang C. Enam S. A. Klein W. L. (1995). Constitutive Alzheimer's-type tau epitopes in a neuritogenic rat CNS cell line. Neurobiol. Aging 16, 583–589. doi: 10.1016/0197-4580(95)00042-D, 8544909

[ref37] Lambert M. P. Stevens G. Sabo S. Barber K. Wang G. Wade W. . (1994). Beta/A4-evoked degeneration of differentiated SH-SY5Y human neuroblastoma cells. J. Neurosci. Res. 39, 377–385. doi: 10.1002/jnr.490390404, 7533843

[ref38] Large T. H. Lambert M. P. Cohen N. M. Klein W. L. (1986). Autonomous control of phosphatidylinositol turnover by histamine and acetylcholine receptors in the N1E-115 neuron-like cell line. Neurosci. Lett. 66, 31–38. doi: 10.1016/0304-3940(86)90161-8, 3714112

[ref39] MacDonald R. C. Ashley G. W. Shida M. M. Rakhmanova V. A. Tarahovsky Y. S. Pantazatos D. P. . (1999a). Physical and biological properties of cationic triesters of phosphatidylcholine. Biophys. J. 77, 2612–2629. doi: 10.1016/S0006-3495(99)77095-5, 10545361 PMC1300535

[ref40] MacDonald R. C. Rakhmanova V. A. Choi K. L. Rosenzweig H. S. Lahiri M. K. (1999b). O-ethylphosphatidylcholine: a metabolizable cationic phospholipid which is a serum-compatible DNA transfection agent. J. Pharm. Sci. 88, 896–904. doi: 10.1021/js990006q, 10479351

[ref41] Mammeri N. E. Dregni A. J. Duan P. Hong M. (2024). Structures of AT8 and PHF1 phosphomimetic tau: insights into the posttranslational modification code of tau aggregation. Proc. Natl. Acad. Sci. USA 121:e2316175121. doi: 10.1073/pnas.2316175121, 38408247 PMC10927509

[ref42] Martin H. Lambert M. P. Barber K. Hinton S. Klein W. L. (1995). Alzheimer's-associated phospho-tau epitope in human neuroblastoma cell cultures: up-regulation by fibronectin and laminin. Neuroscience 66, 769–779. doi: 10.1016/0306-4522(94)00566-N, 7651608

[ref43] Medeiros R. Baglietto-Vargas D. LaFerla F. M. (2011). The role of tau in Alzheimer's disease and related disorders. CNS Neurosci. Ther. 17, 514–524. doi: 10.1111/j.1755-5949.2010.00177.x, 20553310 PMC4072215

[ref44] Miller T. W. Messer A. (2005). Intrabody applications in neurological disorders: progress and future prospects. Mol. Ther. 12, 394–401. doi: 10.1016/j.ymthe.2005.04.003, 15964243

[ref45] Mueller R. L. Combs B. Alhadidy M. M. Brady S. T. Morfini G. A. Kanaan N. M. (2021). Tau: A Signaling Hub Protein. Front. Mol. Neurosci. 14:647054. doi: 10.3389/fnmol.2021.647054, 33815057 PMC8017207

[ref46] Oh J. E. Karlmark K. R. Shin J. H. Pollak A. Freilinger A. Hengstschlager M. . (2005). Differentiation of neuroblastoma cell line N1E-115 involves several signaling cascades. Neurochem. Res. 30, 333–348. doi: 10.1007/s11064-005-2607-2, 16018577

[ref47] Okafor M. Schmitt D. Ory S. Gasman S. Hureau C. Faller P. . (2025). The different cellular entry routes for drug delivery using cell penetrating peptides. Biol. Cell. 117:e70012. doi: 10.1111/boc.70012, 40490965 PMC12149500

[ref48] Otvos L. Jr. Feiner L. Lang E. Szendrei G. Goedert M. Lee V. M. Y. (1994). Monoclonal antibody PHF-1 recognizes tau protein phosphorylated at serine residues 396 and 404. J. Neurosci. Res. 39, 669–673. doi: 10.1002/jnr.490390607, 7534834

[ref49] Pantazatos D. P. MacDonald R. C. (1999). Directly observed membrane fusion between oppositely charged phospholipid bilayers. J. Membr. Biol. 170, 27–38. doi: 10.1007/s002329900535, 10398758

[ref50] Parra Bravo C. Naguib S. A. Gan L. (2024). Cellular and pathological functions of tau. Nat. Rev. Mol. Cell Biol. 25, 845–864. doi: 10.1038/s41580-024-00753-9, 39014245

[ref51] Pope W. Enam S. A. Bawa N. Miller B. E. Ghanbari H. A. Klein W. L. (1993). Phosphorylated tau epitope of Alzheimer's disease is coupled to axon development in the avian central nervous system. Exp. Neurol. 120, 106–113. doi: 10.1006/exnr.1993.1044, 7682967

[ref52] Pope W. B. Lambert M. P. Leypold B. Seupaul R. Sletten L. Krafft G. . (1994). Microtubule-associated protein tau is hyperphosphorylated during mitosis in the human neuroblastoma cell line SH-SY5Y. Exp. Neurol. 126, 185–194. doi: 10.1006/exnr.1994.1057, 7925819

[ref53] Qiu R. MacDonald R. C. (1994). A metastable state of high surface activity produced by sonication of phospholipids. Biochim. Biophys. Acta 1191, 343–353. doi: 10.1016/0005-2736(94)90185-6, 8172920

[ref54] Rawat P. Sehar U. Bisht J. Selman A. Culberson J. Reddy P. H. (2022). Phosphorylated tau in Alzheimer's disease and other Tauopathies. Int. J. Mol. Sci. 23:12841. doi: 10.3390/ijms232112841, 36361631 PMC9654278

[ref55] Rodrigues Martins D. Sha F. Van der Elst W. Shih P. Y. Devoght J. Van Kolen K. . (2023). Anti-tau intrabodies: from anti-tau immunoglobulins to the development of functional scFv intrabodies. Mol. Ther. Methods Clin. Dev. 31:101158. doi: 10.1016/j.omtm.2023.101158, 38074413 PMC10709200

[ref56] Roger P. P. Rickaert F. Huez G. Authelet M. Hofmann F. Dumont J. E. (1988). Microinjection of catalytic subunit of cyclic AMP-dependent protein kinase triggers acute morphological changes in thyroid epithelial cells. FEBS Lett. 232, 409–413. doi: 10.1016/0014-5793(88)80781-6, 2837413

[ref57] Rols M. P. Delteil C. Golzio M. Dumond P. Cros S. Teissie J. (1998). In vivo electrically mediated protein and gene transfer in murine melanoma. Nat. Biotechnol. 16, 168–171. doi: 10.1038/nbt0298-168, 9487524

[ref58] Shim G. Lee S. Kim Y. B. Kim C. W. Oh Y. K. (2011). Enhanced tumor localization and retention of chlorin e6 in cationic nanolipoplexes potentiate the tumor ablation effects of photodynamic therapy. Nanotechnology 22:365101. doi: 10.1088/0957-4484/22/36/365101, 21841215

[ref59] Silinsky E. M. Watanabe M. Redman R. S. Qiu R. Hirsh J. K. Hunt J. M. . (1995). Neurotransmitter release evoked by nerve impulses without Ca2+ entry through Ca2+ channels in frog motor nerve endings. J. Physiol. 482, 511–520. doi: 10.1113/jphysiol.1995.sp020536, 7738845 PMC1157778

[ref60] Slastnikova T. A. Ulasov A. V. Rosenkranz A. A. Sobolev A. S. (2018). Targeted intracellular delivery of antibodies: the state of the art. Front. Pharmacol. 9:1208. doi: 10.3389/fphar.2018.01208 PMC6207587 30405420

[ref61] Smith L. M. Kelleher N. L. Consortium for Top Down, P (2013). Proteoform: a single term describing protein complexity. Nat. Methods 10, 186–187. doi: 10.1038/nmeth.2369, 23443629 PMC4114032

[ref62] Spiller D. G. Giles R. V. Grzybowski J. Tidd D. M. Clark R. E. (1998). Improving the intracellular delivery and molecular efficacy of antisense oligonucleotides in chronic myeloid leukemia cells: a comparison of streptolysin-O permeabilization, electroporation, and lipophilic conjugation. Blood 91, 4738–4746. doi: 10.1182/blood.V91.12.4738, 9616172

[ref63] Stevens G. R. Zhang C. Berg M. M. Lambert M. P. Barber K. Cantallops I. . (1996). CNS neuronal focal adhesion kinase forms clusters that co-localize with vinculin. J. Neurosci. Res. 46, 445–455. doi: 10.1002/(SICI)1097-4547(19961115)46:4<445::AID-JNR6>3.0.CO;2-G, 8950704

[ref64] Takashima A. (2010). Tau aggregation is a therapeutic target for Alzheimer's disease. Curr. Alzheimer Res. 7, 665–669. doi: 10.2174/156720510793611600 20678070

[ref65] Vijayalakshmi A. Prabhakar V. (2026). Pathogenic protein post-translational modifications in Alzheimer's disease: mechanisms and therapeutic strategies. CNS Neurol. Disord. Drug Targets. doi: 10.2174/0118715273448693251223103034, 41944134

[ref66] Wang L. MacDonald R. C. (2011). Cationic phospholiposomes: efficient delivery vehicles of anticancer derivatives of ATP to multiple myeloma cells. J. Liposome Res. 21, 306–314. doi: 10.3109/08982104.2011.565476, 21457078

[ref67] Wang Y. Mandelkow E. (2016). Tau in physiology and pathology. Nat. Rev. Neurosci. 17, 5–21. doi: 10.1038/nrn.2015.1, 26631930

[ref68] Wang H. H. Tsourkas A. (2019). Cytosolic delivery of inhibitory antibodies with cationic lipids. Proc. Natl. Acad. Sci. USA 116, 22132–22139. doi: 10.1073/pnas.1913973116, 31611380 PMC6825285

[ref69] Wojciak-Stothard B. Denyer M. Mishra M. Brown R. A. (1997). Adhesion, orientation, and movement of cells cultured on ultrathin fibronectin fibers. In Vitro Cell. Dev. Biol. Anim. 33, 110–117. doi: 10.1007/s11626-997-0031-4, 9081218

[ref70] Xu T. Su T. Soye B. J. D. Kandi S. Huang C. F. Wilkins J. T. . (2025). The Proteoform landscape of tau from the human brain. J. Proteome Res. 24, 2916–2925. doi: 10.1021/acs.jproteome.5c00139, 40395051 PMC12337710

[ref71] Yoshida H. Ihara Y. (1993). Tau in paired helical filaments is functionally distinct from fetal tau: assembly incompetence of paired helical filament-tau. J. Neurochem. 61, 1183–1186. doi: 10.1111/j.1471-4159.1993.tb03642.x 8360683

[ref72] Zhang C. Lambert M. P. Bunch C. Barber K. Wade W. S. Krafft G. A. . (1994). Focal adhesion kinase expressed by nerve cell lines shows increased tyrosine phosphorylation in response to Alzheimer's a beta peptide. J. Biol. Chem. 269, 25247–25250. doi: 10.1016/S0021-9258(18)47238-9, 7929215

[ref73] Zhang C. Qiu H. E. Krafft G. A. Klein W. L. (1996a). A beta peptide enhances focal adhesion kinase/Fyn association in a rat CNS nerve cell line. Neurosci. Lett. 211, 187–190. doi: 10.1016/0304-3940(96)12761-0, 8817572

[ref74] Zhang C. Qiu H. E. Krafft G. A. Klein W. L. (1996b). Protein kinase C and F-actin are essential for stimulation of neuronal FAK tyrosine phosphorylation by G-proteins and amyloid beta protein. FEBS Lett. 386, 185–188. doi: 10.1016/0014-5793(96)00435-8 8647278

[ref75] Zhu C. Mu J. Liang L. (2024). Nanocarriers for intracellular delivery of proteins in biomedical applications: strategies and recent advances. J. Nanobiotechnol. 22:688. doi: 10.1186/s12951-024-02969-5, 39523313 PMC11552240

